# The Effect of BeBo^®^ Training and EMG-Biofeedback-Assisted Therapy on Pelvic Floor Muscle Function in Women After Vaginal Delivery and Cesarean Section—A Randomized Controlled Trial

**DOI:** 10.3390/jcm14197099

**Published:** 2025-10-08

**Authors:** Paulina Handzlik-Waszkiewicz, Iwona Sulowska-Daszyk, Agnieszka Suder

**Affiliations:** 1 Department of Anatomy, Faculty of Physical Rehabilitation, University of Physical Culture in Cracow, 31-571 Cracow, Poland; 2 Institute of Clinical Rehabilitation, University of Physical Culture in Cracow, 31-571 Cracow, Poland; iwona.sulowska@awf.krakow.pl

**Keywords:** cesarean section, EMG, neuromuscular activity, pelvic floor muscles, vaginal delivery

## Abstract

**Background/Objectives**: Pelvic floor muscle (PFM) training is widely recommended during pregnancy and postpartum as both a preventive and therapeutic intervention. The addition of electromyography (EMG) biofeedback may further enhance training effectiveness. This study aimed to evaluate the effectiveness of a 6-week BeBo^®^ PFM training program (BeBo^®^, derived from the German *Beckenboden*, “pelvic floor”) and to compare it with BeBo^®^ training combined with EMG biofeedback in women after vaginal delivery (VD) and cesarean section (CS), relative to control groups. **Methods:** A total of 120 primiparous women (mean age 29.0 ± 1.9 years), 6–8 weeks postpartum after VD (n = 60) or CS (n = 60), were randomly assigned to BeBo^®^ training, BeBo^®^+EMG-biofeedback, or control group. Neuromuscular PFM activity was assessed before and after intervention using surface electromyography (sEMG; Noraxon G2 TeleMyo 2400) with a vaginal probe. **Results:** Women performing BeBo^®^ training with EMG-biofeedback demonstrated significantly greater improvements in PFM endurance (*p* < 0.001, η^2^ = 0.423) in both VD and CS groups compared with controls and BeBo^®^ training alone. Regarding maximal fast contraction, significant improvements (*p* = 0.042, η^2^ = 0.097) were observed in both delivery groups within the EMG-biofeedback group, whereas BeBo^®^ training alone was effective only in the VD group. **Conclusions:** PFM training based on the BeBo^®^ concept, particularly when supported by EMG-biofeedback, effectively enhances neuromuscular function, with the greatest benefits observed in women after VD. EMG-biofeedback should be considered as an adjunct in standard postpartum preventive care, with training protocols tailored to delivery mode: relaxation-focused for CS and strengthening-focused for VD.

## 1. Introduction

It is estimated that over 200 million women worldwide are unaware of pelvic floor muscle exercises or their effects. Furthermore, 50% of women are unable to perform them correctly [1]. The intimate location of the pelvic floor muscles causes their training to be frequently omitted from general physical activity, despite proven effectiveness in the treatment and prevention of stress urinary incontinence [2].

The pelvic floor is a complex of muscles and fascia stretching between the pubic symphysis, coccyx, and ischial tuberosities [3,4,5]. These structures are arranged in three layers: the superficial layer, the middle layer (urogenital diaphragm), and the deep layer (pelvic diaphragm) [6,7,8]. Pelvic floor muscles play functions essential for health and performance—they maintain urinary, fecal, and gas continence, support the pelvic organs, participate in the regulation of intra-abdominal pressure, and play a role in sexual function and childbirth. Their proper activation ensures core stability, balance, and efficient locomotion [9,10,11].

Pregnancy and childbirth are considered the main factors weakening the pelvic floor muscles [12]. Perinatal factors that may impair the pelvic floor include: neonatal head circumference above 38 cm, birth weight over 4 kg, prolonged second stage of labor, vacuum-assisted or forceps delivery, episiotomy, and perineal tissue injury [13,14]. It is widely believed that cesarean section carries a lower risk of pelvic floor dysfunction compared to vaginal delivery. However, studies have shown that a cesarean section performed in the second stage of labor does not prevent disruption of pelvic floor muscle integrity to the same extent as a cesarean section performed in the first stage or electively [15,16].

The postpartum period refers to the time following childbirth during which both the reproductive organs and systemic adaptations associated with pregnancy and delivery gradually return to their pre-pregnancy state [17]. This period typically lasts between 6 and 8 weeks. Traditionally, the postpartum period is divided into three phases: Immediate postpartum—the initial 24 h after delivery, Early postpartum–the first week following delivery, Late postpartum—extending up to 6 weeks after delivery [18].

The BeBo^®^ method is a holistic pelvic floor muscle training program applied in urological and gynecological prevention and rehabilitation [19]. It comprises five groups of exercises: awareness, mobilization, strengthening, relaxation, and integration into daily activities [20]. Special emphasis is placed on correct posture, breathing, and movement ergonomics, as well as daily habits and scar mobilization [21,22].

The method of biological feedback (biofeedback) enables patients to observe muscle activity in real time [23]. The most commonly used tool is surface electromyography (sEMG), which non-invasively records the bioelectrical signals of muscles [24]. The use of biofeedback allows for proper activation of pelvic floor muscles, control of contraction strength and duration, and enhances patients’ awareness and motivation [2,25,26]. It is crucial to limit the involvement of synergistic muscles so as not to disturb intra-abdominal pressure [27].

Previous studies on pelvic floor muscle training, including BeBo^®^ training, have rarely addressed issues resulting from pelvic floor weakness in women after cesarean section, treating them as considerably less exposed to dysfunction within this muscle group. The topic of comparing the effectiveness of the BeBo^®^ method and the BeBo^®^ method supported by EMG-biofeedback has not been widely described. As indicated in the literature, it is also necessary to consider to what extent supportive methods increase the effectiveness of pelvic floor muscle therapy.

The aim of this study was to evaluate the effectiveness and compare the outcomes achieved during a 6-week training program based on the BeBo^®^ concept and BeBo^®^ training supplemented with a 10 min EMG-biofeedback session on pelvic floor muscle (PFM) function in women after vaginal delivery and cesarean section, compared to control groups.

Accordingly, it was hypothesized that training according to the BeBo^®^ concept, especially BeBo^®^ training supported by EMG biofeedback, leads to improved neuromuscular activity of the pelvic floor muscles (PFM), assessed by sEMG examination, in women after natural childbirth and cesarean section compared to control groups.

## 2. Materials and Methods

### 2.1. Study Design

The study was conducted at the Department of Anatomy and in the Functional Diagnostics Laboratory of the Central Scientific Research Laboratory at the University of Physical Culture in Cracow. It was a single-blind, randomized controlled trial conducted in accordance with the Declaration of Helsinki. Ethical approval was obtained from the Bioethics Committee (approval number 69/KBL/OIL/2021).

The project was funded under University of Physical Education in Cracow grant number 148/MN/INP/2021 and was registered on the Australian New Zealand Clinical Trials Registry (ACTRN12622000563763). The study followed the CONSORT guideline for randomized controlled trials.

### 2.2. Participants

The study included women aged 25–35 years, 6–8 weeks after their first delivery (either vaginal or cesarean). Inclusion criteria included: female sex, first delivery, physiological course of pregnancy, provision of written informed consent, and no medical contraindications. Exclusion criteria were: lack of consent or medical certificate, multiple pregnancy, more than one delivery, postpartum complications (pubic symphysis diastasis, thrombophlebitis, third–fourth degree perineal tears, abdominal or reproductive system surgeries), severe orthopedic conditions, stage III–IV pelvic organ prolapse, urinary tract or vaginal infections, perineal sensory disorders, endometriosis, and other diseases that could hinder participation or affect the results (including thyroid disorders, diabetes, obesity, cancers, rheumatic, respiratory, neurological, cognitive, and mental disorders).

**Figure 1:**
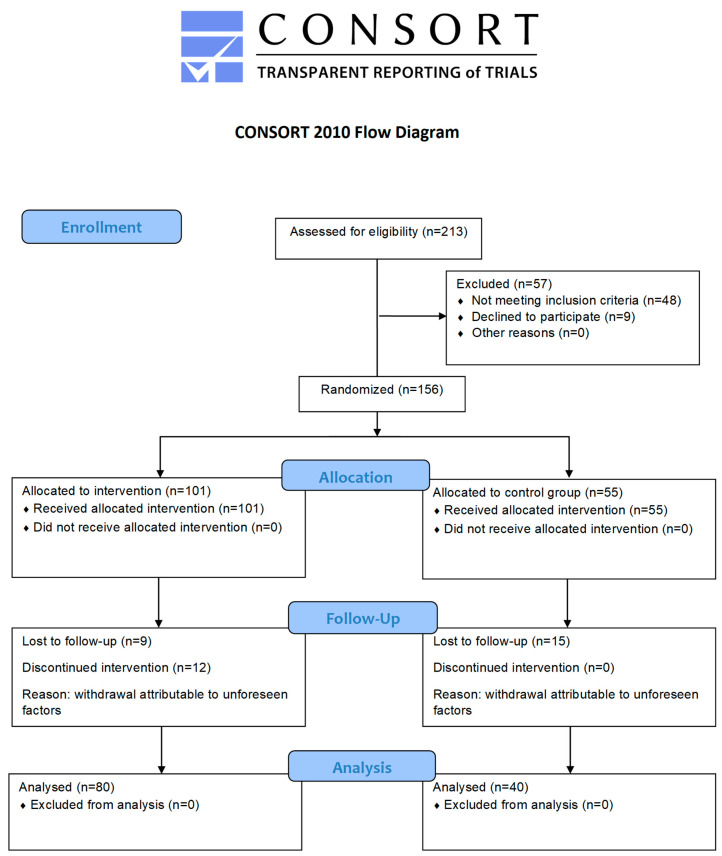
CONSORT flow diagram.

Recruitment of participants took place from 12 April 2022 to 18 September 2023 through a social media announcement, gynecologists and midwives from Cracow hospitals, as well as informational materials (leaflets and posters). Participants were enrolled during pregnancy. After childbirth, each woman underwent a gynecological examination to evaluate potential contraindications for inclusion in the study. All measurements were completed by 18 December 2023. The study flow diagram is presented in Figure 1.

This study was a parallel-group, randomized, controlled trial with a 1:1:1 allocation ratio. Following gynecological consultation and verification of the inclusion and exclusion criteria, the cohort of women who delivered either vaginally or via cesarean section was randomly allocated to one of the following three study groups: vaginal delivery (VD)–BeBo^®^ training (I), BeBo^®^+EMG-biofeedback training (II), control group (III), cesarean section (CD)–BeBo^®^ training (IV), BeBo^®^+EMG-biofeedback training (V), control group (VI).

**Figure 2:**
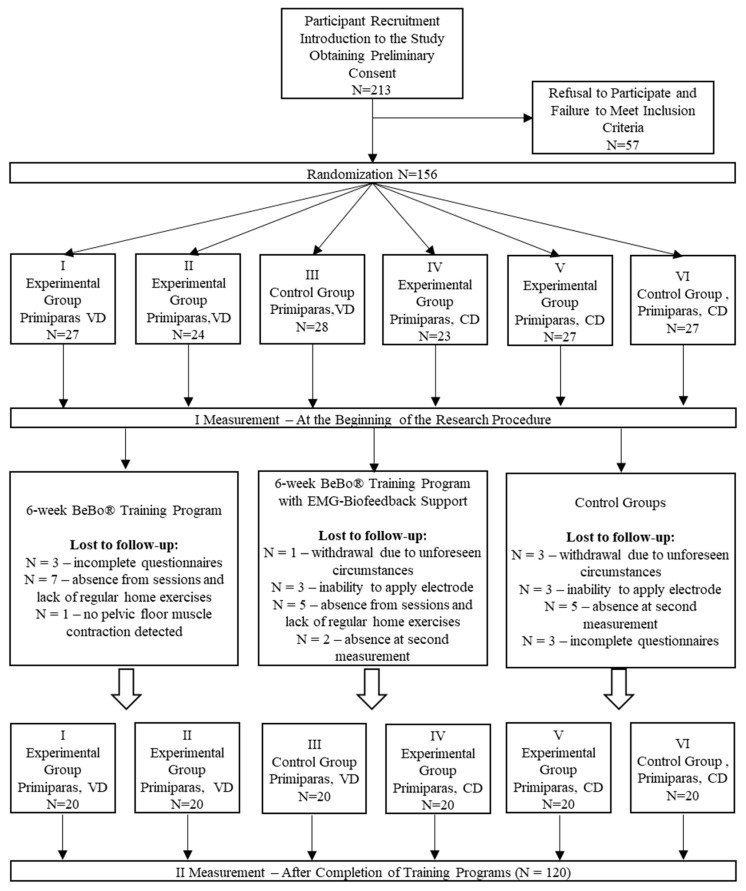
Study flow diagram. VD—vaginal delivery, CD—cesarean section, N—number of participants.

Randomization was performed using a computer-generated random number generator [GraphPad] by a member of the research team who did not participate in the patient recruitment process. A total of 213 women were recruited for the study, of whom 156 began participation after gynecological qualification. During the course of the study, 36 participants were excluded, mainly due to exceeding the maximum of two permissible absences from training sessions. Ultimately, 120 women completed the training program. A detailed qualification scheme is presented in Figure 2.

The groups were homogeneous in terms of age (29.0 ± 1.9 years), body mass (67.9 ± 8.8 kg), height (165.8 ± 5.8 cm), and BMI (24.7 ± 2.6 kg/m^2^). Detailed characteristics are presented in Table 1.

### 2.3. Research Tools

Participants, regardless of group allocation, underwent measurements at baseline and after six weeks of observation (control groups) or intervention: BeBo^®^ training and BeBo^®^ training supported with EMG-biofeedback. Before commencing the study, participants were informed about the purpose and procedure of the research and therapy, and they provided written consent to participate in the research project. Subsequently, direct anthropometric measurements were taken: body height and body mass were measured. Instruction was provided regarding the anatomy and function of the pelvic floor. Participants were taught correct activation of the pelvic floor muscles without engaging the gluteal, abdominal, or adductor muscles. The instruction also included teaching proper breathing during pelvic floor muscle contraction. Pelvic floor muscle activity was measured using surface electromyography. The researcher who performed the measurements was blinded to the participant group allocation.

#### Surface Electromyography (sEMG)

**Figure 3:**
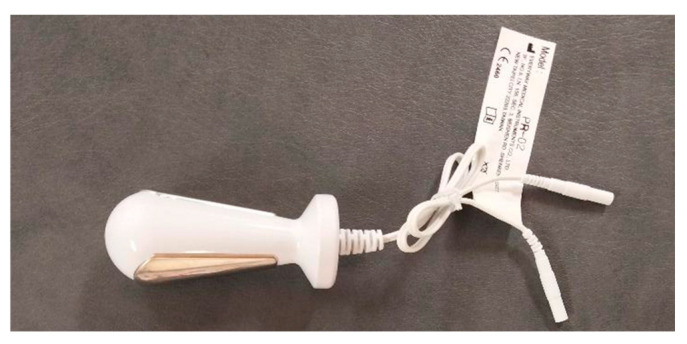
Vaginal probe Lifecare PR-02 (Everyway Medical Instruments Co., Ltd., Taiwan).

Neuromuscular activity of the pelvic floor muscles was assessed using the Noraxon Ultium device (Noraxon U.S.A. Inc., Scottsdale, AZ, USA) and a vaginal probe (Lifecare PR-02, Everyway Medical Instruments Co., Ltd., New Taipei City, Taiwan) (Figure 3) with the surface electromyography (sEMG) method. The signal was recorded with a 9-channel system with 16-bit resolution and a sampling frequency of 1500 Hz. The vaginal probe, 76 mm in length and 28 mm in diameter, was composed of two longitudinal plates made of stainless steel and nickel. Measurements were not performed during bleeding associated with uterine involution or during menstruation.

Electromyographic data were band-pass filtered within the range of 10–500 Hz, rectified, and smoothed using RMS filtering with a 100 ms window, and expressed in microvolts (µV) and as a function of frequency (Hz). In addition, sEMG data were normalized to the maximum recorded RMS EMG amplitude for the pelvic floor muscles during a series of maximal contractions.

All sEMG examinations were conducted in the same laboratory, in the afternoon, between 16:00 and 21:00. Room temperature was maintained between 20–22 °C. The measurements were performed by the same physiotherapist, experienced in urogynecology and trained in the use of sEMG.

Prior to measurement, participants were asked to empty their bladder. Subsequently, wearing gloves and applying gel, each participant inserted the electrode herself, positioning the plates laterally toward the hips. The correct placement of the electrode was verified by the physiotherapist. Halski et al. [28] observed that different probe placements during functional pelvic floor muscle contraction do not affect the results obtained with sEMG. The examination was carried out in the supine position. A roller was placed under the knees so that the hip and knee joints were slightly flexed. The spine was kept in a neutral position, and the upper limbs were placed alongside the trunk. The sEMG signal was processed using the MyoResearch XP 3.14 software (Noraxon U.S.A. Inc., Scottsdale, AZ, USA). The vaginal electrode could not dislocate during pelvic floor muscle contraction and was not allowed to cause pain or discomfort.

**Figure 4:**
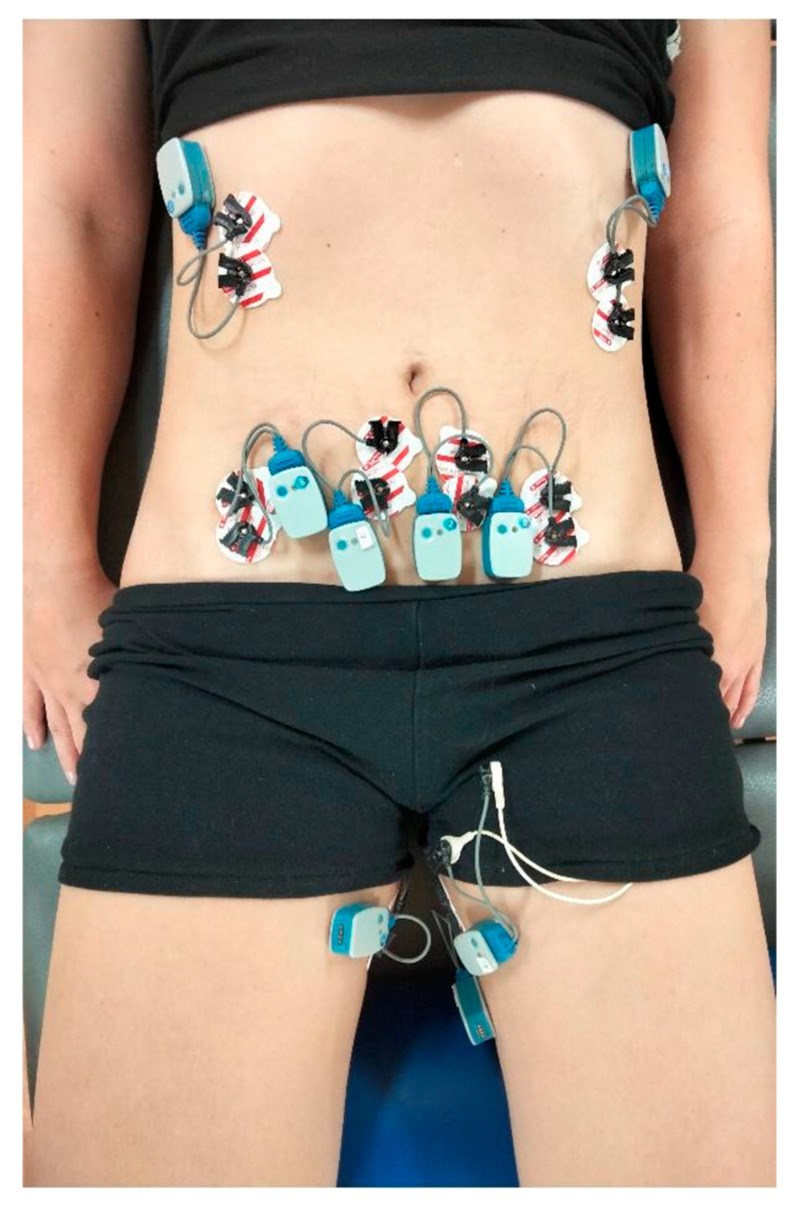
Placement of surface electrodes during the examination.

Surface electrodes (Sorimex, Toruń, Poland) were placed in accordance with SENIAM project guidelines [29] (Figure 4). The skin at electrode sites was cleaned with salicylic alcohol to improve adhesion and reduce the probability of signal artifacts. Electrodes were positioned at a distance of 2 cm (center-to-center) along the muscle fiber orientation on the following muscles: adductor longus, rectus abdominis, internal oblique, and external oblique (Figure 4). The signals from these muscles were recorded before testing pelvic floor muscles to ensure that participants learned proper PFM activation without engaging synergistic muscles.

The study was conducted according to the protocol proposed by Oleksy et al. [30], consisting of five activities:

1. 60 s rest (before starting the test—divided into 3 intervals: I—5 s, II—5 s, III—50 s)–during this phase, participants were instructed to feel pelvic floor muscle relaxation.
2. Five 2 s phasic contractions (quick movements), with 10 s rest intervals—participants were instructed to contract the pelvic floor muscles as quickly as possible, then rapidly and completely relax them.
3. Five 10 s tonic contractions, with 10 s rest intervals—participants were instructed to contract the pelvic floor muscles as strongly as possible, maintain the contraction for 10 s, and then completely relax.
4. One 60 s endurance contraction—participants were instructed to contract the pelvic floor muscles at a submaximal level that they could maintain for 60 s without changing the contraction intensity.
5. 60 s rest (after completing the exercises)—participants were instructed to feel pelvic floor muscle relaxation.

### 2.4. Intervention

#### 2.4.1. Pelvic Floor Muscle Training Program According to the BeBo^®^ Concept

Patients from four study groups (I, II, IV, V) participated for six weeks in individual, one-hour training sessions according to the BeBo^®^ concept, conducted twice weekly by a licensed urogynecological physiotherapist with five years of professional experience. Each session included a 10 min theoretical part and a 50 min practical part. Two absences were permitted during the training period, which had to be compensated for with prescribed home exercises.

Each training unit included: awareness exercises, strengthening exercises, mobilization exercises, integration of pelvic floor muscles into daily activities, and relaxation exercises [22].

Awareness exercises aimed at learning the location, structure, and function of the pelvic floor muscles, as well as orientation to the bony landmarks that serve as muscle attachments. Visualization techniques were used during the sessions. Awareness training also allowed participants to perceive connections between different body regions and the pelvic floor muscles. These regions included the area around the eyes, jaw, interscapular region, and feet [22].

Mobilization exercises included movements of the joints directly connected to the pelvic girdle, primarily the lumbar spine, hip joints, and lumbosacral junction, as well as other body regions connected to the pelvic floor via fascial structures. The purpose of this group of exercises was to increase the flexibility of myofascial structures [22].

The BeBo^®^ method comprises three program variants:

A—mobilization exercises without contracting the pelvic floor muscles,
B—rhythmic contraction and relaxation, including with pelvic movements,
C—prolonged maintenance of pelvic floor muscle contraction [20].

Relaxation exercises aimed at restoring the balance between the sympathetic and parasympathetic nervous systems, thereby calming cardiac and respiratory activity and reducing stress levels [20].

Integration of the pelvic floor muscles into daily activities focused on protecting the perineum by implementing correct habits that prevent overloading of the pelvic floor muscles. The training was designed to teach, among other things, proper techniques for bending, standing up, sitting down, lifting, and carrying the baby or other heavy objects [22].

Special attention was given to teaching correct body posture, breathing exercises, and balance training. Exercises were performed in various starting positions: standing, quadruped, knee–elbow, sitting, supine, side-lying, and prone. Training equipment included: a large and a small ball, a sensorimotor disc, a foam roller, a tennis ball, and cherry pit bags.

#### 2.4.2. Training with EMG-Biofeedback

EMG-biofeedback training was performed using the Noraxon Ultium device (Noraxon U.S.A. Inc., Scottsdale, AZ, USA) after each one-hour BeBo^®^ training session in groups II and V. It consisted of performing isolated contractions and conscious relaxation of the pelvic floor muscles with the aid of visual feedback. The EMG-biofeedback training lasted 10 min and was supervised by the same experienced urogynecological physiotherapist.

Before the training, the participant, wearing gloves and using gel, self-inserted the vaginal electrode with the plates positioned laterally toward the ischial tuberosities; the correct placement was then verified by the physiotherapist. Surface electrodes were subsequently applied to the disinfected skin over the external oblique, internal oblique, rectus abdominis, and adductor muscles according to SENIAM methodology [29].

During training, the participant remained in the supine position with a roller placed under the knees. The upper limbs were positioned alongside the trunk. A laptop screen was placed at the participant’s eye level so that, during pelvic floor muscle contraction, she could observe the displacement of an indicator reflecting muscle tension. The indicator was displayed in the form of an electromyogram.

Before each session, the maximal voluntary contraction (MVC) of the pelvic floor muscles was recorded as the highest value among three performed contractions. The indicator from the vaginal electrode moved upward during correctly performed tasks. If the level of pelvic floor muscle tension decreased before the specified time, the indicator dropped; in the absence of contraction, it did not change position. If the participant was unable to complete a task at the planned level of tension, the task was omitted.

The participant performed the tasks according to the following scheme:

- Pelvic floor muscle activation for 3 s–5 repetitions at 20% MVC;
- Pelvic floor muscle activation for 5 s–5 repetitions at 20% MVC;
- Pelvic floor muscle activation for 10 s–5 repetitions at 20% MVC;
- 1 min rest break;
- Pelvic floor muscle activation for 3 s–5 repetitions at 50% MVC;
- Pelvic floor muscle activation for 5 s–5 repetitions at 50% MVC;
- Pelvic floor muscle activation for 10 s–5 repetitions at 50% MVC;
- 1 min rest break;
- Pelvic floor muscle activation for 3 s–5 repetitions at 80% MVC;
- Pelvic floor muscle activation for 5 s–5 repetitions at 80% MVC;
- Pelvic floor muscle activation for 10 s–5 repetitions at 80% MVC.

### 2.5. Statistical Analysis

Analyses were performed using Statistica 13. Data were described using mean, standard deviation, median, and minimum and maximum values. To assess the effects of intervention type, mode of delivery, and time, repeated measures analysis of variance (ANOVA) was applied. In the case of significant results, Fisher’s LSD post hoc test was performed, and homogeneity of variance was verified with Levene’s test. The significance level was set at *p* < 0.05.

In the post hoc mode, a power analysis for ANOVA was conducted, which showed that with 20 participants in each of the six groups, the study had nearly full power to detect medium effects (99.97% for the main effect, 99.4% for the interaction) and large effects (~100%), but limited power in the case of small effects (58.4% and 33.3%). When test assumptions were not met, additional statistical procedures were applied. Effect size (ES) was calculated using Hedges’ g, which provides greater accuracy in small samples. The effect size results were interpreted as small for g < 0.2, medium for g < 0.5, large for g < 0.8, and very large for g > 0.8.

## 3. Results

### 3.1. Measurements of Neuromuscular Activity of the Pelvic Floor Muscles at Rest

The analysis of changes in surface electromyography (sEMG) measurements began with a comparison of the minimum pelvic floor muscle (PFM) tension during 1 min rest. Repeated measures analysis of variance revealed a significant effect of delivery mode (*p* < 0.001, η^2^ = 0.205) and time (*p* < 0.001, η^2^ = 0.313) on resting tension, as well as significant interactions between time and delivery mode (*p* = 0.015, η^2^ = 0.069), and between time, delivery mode, and training (*p* = 0.048, η^2^ = 0.045).

Post hoc analysis showed a significant decrease in measured tension between pre- and post-intervention assessments in both training groups of women after cesarean section. Further post hoc analysis also revealed a significant difference between training and control groups in the CC group, as well as a significantly greater reduction in tension in the group performing EMG-biofeedback–assisted training compared to the BeBo^®^ training group.

Effect size analysis (pre vs. post) indicated that in the EMG-biofeedback groups the effect was medium to large, in the other training groups it was borderline medium to small, and in the control groups it was small. The results are presented in Table 2.

### 3.2. Measurements of Neuromuscular Activity of the Pelvic Floor Muscles During Maximal Contractions

Both the mean value of five maximal PFM contractions (*p* < 0.001, η^2^ = 0.359) and the measured maximal voluntary contraction (MVC) varied only by delivery mode (*p* < 0.001, η^2^ = 0.336), with both values being significantly higher in the group of women after cesarean section (Table 3). A significantly higher ratio of maximal pelvic floor muscle tension to resting tension was observed at the second measurement point (*p* < 0.001, η^2^ = 0.217), as well as an interaction between time and type of training for this parameter. Post hoc analysis showed that significant temporal changes in this ratio were present only in the training intervention groups. Furthermore, post hoc comparisons between groups at T2 revealed no significant differences between training and control groups among women after vaginal delivery, whereas in women after cesarean section a significant difference was found in the group undergoing EMG-biofeedback–assisted training. The calculated effect size (ES) was also highest in these groups, indicating a large effect.

Analysis of the variable time before peak (TBP) showed a significant decrease between measurements (*p* < 0.001, η^2^ = 0.157) and an interaction between the main factors of time and type of training (*p* = 0.042, η^2^ = 0.097). Detailed post hoc analysis demonstrated that the significant reduction in PFM activation time concerned the training groups. Significant differences were also observed between both training groups and the control group in women after vaginal delivery, as well as between the EMG-biofeedback group and both the control group and the BeBo^®^ training group in women after cesarean section. An effect of delivery mode (*p* < 0.001, η^2^ = 0.159) and time (*p* < 0.001, η^2^ = 0.215) was also observed for resting PFM tension between maximal contractions. The data are presented in Table 3.

### 3.3. Measurements of Neuromuscular Activity of the Pelvic Floor Muscles During 10 s Tonic Contractions

When analyzing neuromuscular activity during 10 s tonic contractions, an increase in contraction amplitude was observed in all groups; however, these changes were not significant with respect to the overall effects of time or type of training. Detailed post hoc analysis, however, revealed a significantly higher contraction amplitude in the CC group performing EMG-biofeedback–assisted training (*p* = 0.032). Nevertheless, the calculated ES indicated a small to very small effect (Table 4). Differences were noted depending on the mode of delivery (*p* < 0.001), as tension in the groups of women after cesarean section was significantly higher, reaching more than twice the values. Resting tension between contractions was also analyzed. Significantly lower resting amplitudes were observed in women after vaginal delivery. Women after CC reached mean values above 10 µV, whereas the mean result for women after vaginal delivery was approximately 6 µV. However, in terms of maximal contraction (MVC), women after vaginal delivery achieved higher results. A decrease in resting tension over time was also confirmed (*p* < 0.001), including in relation to MVC. Although the decrease was greater in the intervention groups, no significant differences were found in comparison with the control groups. Notably, the ES in the EMG-biofeedback intervention groups was at the threshold between small and medium effect size (Table 4).

### 3.4. Descriptive Statistics and Results of Repeated Measures ANOVA for Parameters Measured During the 60 s Endurance Contraction

Before the intervention, the median frequency values of the signal during the 1 min submaximal isometric contraction in groups I–III and IV–VI were similar. The differences observed in these groups were not statistically significant. No significant differences were found between women after vaginal delivery and those after cesarean section. No consistent trend was noted regarding changes in median frequency after training, and no statistically significant changes were observed for the main effects or their interactions. The calculated ES also did not indicate a substantial effect of changes in frequency (Table 5).

The variable representing motor endurance of the PFM was the ratio of the median frequency during the last 5 s of the 1 min submaximal contraction to the median frequency during the middle 5 s. Due to delayed PFM activation in some participants, the ratio of the last 5 s to the first 5 s was not calculated. No statistically significant differences were found depending on delivery mode. However, analyses indicated that both the effect of time (*p* < 0.001, η^2^ = 0.127) and the interaction between time and type of training (*p* = 0.045, η^2^ = 0.071) significantly influenced changes in this ratio. Further analyses showed that this was particularly evident in the EMG-biofeedback training groups (*p* = 0.008 and *p* = 0.006). Post hoc analysis revealed that these groups achieved significantly higher results after the intervention compared to the control groups. Among women after cesarean section, the EMG-biofeedback group also obtained significantly higher results than the BeBo^®^ training group.

The presence of significant differences was also confirmed by the calculated effect size, which indicated a very large effect for groups II and V. In the training groups I and IV, ES reached medium and very large levels, respectively; however, statistical analysis did not show significant changes.

The mean amplitude of the PFM EMG signal during the 1 min submaximal isometric contraction before and after the training intervention was also analyzed. When evaluating amplitude relative to MVC, no major changes in contraction values were observed. However, in terms of the absolute amplitude values, statistically significant effects were found for time and for the interaction between time and training. Detailed pre vs. post analysis indicated that significant changes concerned women after cesarean section in the training intervention groups (*p* = 0.024 and *p* = 0.007). In contrast, when comparing training groups with control groups, no differences were observed at the assumed level of statistical significance (Table 5).

### 3.5. Measurements of Neuromuscular Activity of the Pelvic Floor Muscles at Rest After Completion of the Study

After completion of the sEMG procedure, resting PFM activity was measured again. In all groups, both before and after training, a decrease in measured tension amplitude was observed compared to the initial resting measurement. Statistical analysis showed a main effect of time (*p* < 0.001, η^2^ = 0.216) resulting in a decrease in measured tension. For this parameter, repeated measures analysis of variance also revealed significant differences between the CC and SN groups (*p* = 0.003). The data are presented in Table 6.

Additionally, PFM neuromuscular activity at rest before and after the first sEMG measurement procedure was compared. Repeated measures analysis of variance demonstrated a significant effect of time (*p* < 0.001, η^2^ = 0.164) and delivery mode (*p* < 0.001, η^2^ = 0.280), as well as a significant interaction between time and delivery mode (*p* < 0.001, η^2^ = 0.171). Detailed post hoc analysis showed that the significant decrease in amplitude concerned only the groups of women after cesarean section. Detailed results are presented in Table 7.

## 4. Discussion

In our study, a decrease in pelvic floor muscle (PFM) resting tone was observed following the implemented training programs. This effect was particularly evident in women who delivered by cesarean section, where the results were significantly better compared with the control group, in which the changes were not statistically significant. The use of EMG-biofeedback-assisted training, based on performing correct PFM activation at a predetermined level for a defined period of time, proved to be more effective than BeBo^®^ training alone, allowing for greater PFM relaxation. Assessment of resting activities between subsequent tasks also demonstrated that, over time, the ability of the pelvic floor muscles to relax improved. Furthermore, measurements of resting activity taken after completion of the study revealed lower amplitude values of muscle tone compared with the initial measurements at full relaxation, suggesting that even the activities associated with the study itself may have contributed to PFM relaxation.

The obtained results are consistent with two different studies conducted by Chmielewska et al. [31,32]. In the first study, 21 healthy nulliparous women aged 19–28 years were enrolled in a six-week EMG-biofeedback training program. Surface electromyography measurements were performed four times according to the Glazer protocol [33]: at baseline, three weeks after training, six weeks after training, and one month after its completion. The exercises were carried out three times per week, with participants alternating between strengthening and endurance training. The authors demonstrated that pelvic floor muscle training assisted with biofeedback significantly reduced resting pelvic floor muscle activity in both supine and standing positions (by approximately 10% MVC), and the ability to relax the pelvic floor muscles after a 60 s contraction improved markedly in both positions [31].

In another study conducted in two groups of women with stress urinary incontinence, the team of Chmielewska [32] obtained similar results. The women participated in different training programs: EMG-biofeedback training and Pilates training. In both cases, as in our study, a reduction in resting bioelectrical activity of the muscles was observed; however, this decrease was statistically significant only in the EMG-biofeedback group. In our study, such changes were noted in the groups of women after cesarean section who underwent BeBo^®^ training as well as in the group receiving EMG-biofeedback-assisted training, with the latter showing a significantly greater effect.

In both projects, the team of Chmielewska [31,32] also compared the normalized amplitude of resting activity following a 60 s contraction. Their results indicated that EMG-biofeedback-assisted training enhanced the ability to achieve greater relaxation. This is fully consistent with our findings, in which a reduction in PFM activity after a 60 s contraction was also observed in both groups exercising with EMG-biofeedback. It is assumed that performing PFM contractions may serve as a relaxation technique, leading to a reduction in vaginal pressure and resting PFM activity [34].

In addition to evaluating the effectiveness of the training programs compared with the control groups, the present project also identified differences in PFM activity and functionality depending on the mode of delivery. When assessing neuromuscular activity both at rest and during maximal contraction, significant differences were observed between the groups. Resting tone was found to be nearly twice as high in women who delivered by cesarean section. PFM tone after cesarean delivery may be altered due to the surgical incision through the abdominal muscles and fascia, which affects coordination and intra-abdominal pressure control. For this reason, some women may experience increased pelvic floor muscle tension as a defensive mechanism in response to pain and discomfort around the postoperative scar [35,36]. The difference may also result from the fact that women delivering vaginally are more exposed to both stretching and/or tissue damage during childbirth [12,13].

In our study, EMG assessment of both the mean values of maximal voluntary contractions (MVC) and the highest individual contraction revealed an increase in mean amplitude; however, this change was not statistically significant. By contrast, when comparing the ratio of MVC to resting tone, significantly favorable results were observed in the training groups. Analysis of the training effects relative to the control groups showed that the best outcomes were obtained in the cohort of women after cesarean section who exercised with EMG-biofeedback assistance. This may be attributed to the acquired ability to consciously and deliberately perform PFM contractions.

Improvement in neuromuscular activity and an increase in EMG amplitude during rapid maximal contractions were also reported by Szumilewicz et al. [37] in their study evaluating the effects of a 6-week group training program consisting high-intensity aerobics and PFM exercises in pregnant women.

A systematic review and meta-analysis conducted by Wu et al. [38] demonstrated that the use of EMG-biofeedback in PFM training enhances the effectiveness of pelvic floor therapy. A large proportion of the cited studies indicate a greater increase in PFM strength or measured amplitude of muscle activity.

According to Błudnicka et al. [39], even a single session of EMG-biofeedback significantly improves contraction awareness. Their study showed that one biofeedback session markedly enhanced PFM contractions in pregnant women. In the biofeedback group, more women maintained or improved correct technique compared with the control group (73% vs. 65%). Statistically significant improvements in the sequence of PFM activation were observed in four tasks: the first, third, and fifth rapid contractions, as well as the first 10 s contraction. In the control group, improvement was noted in only one motor task. A single EMG-biofeedback session should be recommended for pregnant women without urinary incontinence to teach them the correct performance of PFM exercises.

In addition to resting tone, women who delivered by cesarean section also achieved significantly higher results during maximal contractions. The literature reports greater PFM activity in women after cesarean delivery, which may be attributed to the absence of tissue trauma during childbirth. Pereira et al. [40] conducted a study in a cohort of 384 women, assessing PFM using surface electromyography, palpation, and urinary incontinence questionnaires. The greatest ability to recruit motor units during PFM contraction was observed in nulliparous women, followed by primiparas, women after cesarean section, women after vaginal delivery, women in the climacteric period, and postmenopausal women. As a result of musculoskeletal changes occurring during pregnancy, women in the postpartum period are characterized by increased lumbar lordosis. In the study by Capson et al. [41], it was noted that women with a hyperlordotic posture exhibited lower PFM activity. The BeBo^®^ concept emphasizes teaching correct body posture in various daily activities and additionally focuses on performing posterior pelvic tilts in different body positions, which may contribute to improved PFM activation. An important parameter describing muscle functionality and training level is the time to reach peak contraction. In the case of rapid maximal PFM contractions, which largely activate fast-twitch fibers, a reduction in the time to peak contraction indicates improved activation [42]. In the context of urinary incontinence, this may be of key importance for urethral closure at moments of increased intra-abdominal pressure, such as during sneezing or coughing [43].

Over time and with training, a statistically significant reduction in the interval from the onset to the peak of contraction was observed in all training groups, whereas no such changes were demonstrated in the control groups. Comparative analysis further indicated that in women after VD, the outcomes achieved in the training groups were significantly better compared with the non-intervention group. An interesting finding was obtained in the cohort of women after CD, where this relationship was observed only among those exercising with biofeedback assistance. Moreover, the outcomes achieved in this group were significantly superior to those of women training according to the BeBo^®^ concept alone.

According to the present study, there was no statistically significant difference in the time from contraction onset to peak between women after VD and those after CD, although the latter demonstrated generally lower TBP values.

From the perspective of PFM functionality, the analysis of 10 s tonic contractions and 1 min sustained endurance contractions is of particular importance, as they provide valuable information regarding PFM endurance. When comparing the mean amplitude of tonic contractions, a significant difference was observed between women in the VD and CD groups. Women after CD achieved higher absolute values, although the percentage of MVC remained comparable between the two groups.

The analysis of the effects of time and training on the obtained results demonstrated a significant increase in maximal amplitude during the 1 min sustained contraction in the CD training groups. However, these changes were not statistically significant in relation to the outcomes obtained in the control groups. Similar findings were reported by Szumilewicz et al. [37], who did not observe significant increases in amplitude during longer 1 min contractions.

The measured amplitude distinguished women who delivered by CD from those after VD. The significantly lower amplitude observed in the latter group, apart from the overall PFM load associated with vaginal delivery, may also be attributed to episiotomy or perineal tears, which in our study affected well over half of the participants. A similar association was indicated by Min et al. [44], who demonstrated that women with a history of episiotomy exhibited significantly lower EMG signal amplitudes during both maximal and endurance contractions.

Therefore, from the perspective of endurance assessment, the evaluation of the median frequency of the EMG signal appears to be of greater relevance. For this purpose, both the mean median frequency of the entire contraction and the changes in median frequency over the course of the contraction were analyzed. In the first case, no significant differences were observed either before or after training, nor between the VD and CD groups.

When analyzing changes in the median frequency, a difference was observed, particularly in the training groups exercising with EMG-biofeedback assistance. Before the intervention, a shift of the median frequency toward lower values was observed in the final seconds of the contraction, whereas after training the values remained stable. The groups utilizing biofeedback achieved significantly better results compared with the control groups, and in women after CD also in comparison with the group training solely according to the BeBo^®^ concept. According to the literature, a shift of the median frequency toward lower values is indicative of muscle fatigue [45]. Therefore, the results obtained in the present study suggest greater resistance to fatigue and improved endurance of the pelvic floor muscles (PFM) following the implementation of training programs.

The analysis of sEMG measurements indicates improved PFM functionality in the groups undergoing training interventions. Positive results, confirmed by effect size analysis, were obtained for many variables both in the groups exercising according to the BeBo^®^ concept and in those following the BeBo^®^ concept supported with EMG-biofeedback. However, when looking more closely at the results, greater improvements were observed in the groups that, in addition to standard training, used the biofeedback technique. This may be the result of several factors. Above all, women training with EMG-biofeedback can simultaneously monitor PFM activation, ensuring the correctness of contraction performance. As noted by Burns et al. [46], the use of biofeedback during pelvic floor muscle exercises improves muscle coordination and control. The application of biofeedback also enables faster response to PFM contractions. This contributes to more effective training, which is reflected in the results obtained [47].

Motivation to exercise must also be considered. The possibility of observing one’s own training in situ, as well as the interaction with the therapist, undoubtedly encourages exercise [48]. This is also a valuable tool for the therapist, who can correct errors in real time—errors that are not always detectable during traditional training.

Another unquestionable advantage of biofeedback-assisted training is the ability to detect very small contraction amplitudes caused by weak muscle force. This is particularly important in sensitive groups of patients, such as postpartum women [49].

The results obtained clearly demonstrated the usefulness of EMG-biofeedback in pelvic floor muscle rehabilitation and training. This raises the question of using feedback devices for patients, for example in the form of mobile applications. In a study by Chu et al. [50], the use of a smartphone application improved PFM activation and, importantly, participants using it were more adherent to training recommendations.

In other studies, the use of audio-based applications demonstrated significantly better PFM training results and greater effectiveness in treating urinary incontinence compared with traditional training methods [51]. Another project employing biofeedback used the Perifit device, and in a group of 6000 women it was shown that such a tool can be useful in the treatment of urinary incontinence [52].

The present study demonstrated the effectiveness of EMG-biofeedback–assisted training methods. Despite the fact that studies exist regarding the effectiveness of different training forms, no standardized protocol has been established that specifies the optimal training duration, number of contractions, number of sets, or body positions for biofeedback-assisted exercises. Bø and Sherburn [53] emphasized that pelvic floor muscle function in women can be significantly improved if a well-designed training program is supported with proper monitoring and feedback. Designing a resistance training program requires consideration of exercise type, frequency, intensity, and training duration. To achieve beneficial adaptive changes, it is also necessary to apply periodization of training volume, load, and intensity.

### Limitations of the Study

Several limitations were identified in the present study. The relatively small sample size may have reduced the ability to detect subtle effects and differences between factors, although the estimation of the minimum required sample size allowed for valid inference. Another limitation was the considerable dropout rate among participants, most likely related to the time demands of the intervention. Young mothers, facing numerous new challenges, were not always able to regularly attend the 6-week training program without sufficient support from their environment. Despite the broad acceptance of sEMG as a method for evaluating PFM activity, the deep anatomical location of the pelvic floor muscles is associated with the risk of signal crosstalk from adjacent muscle groups. The results may have been influenced by factors such as body composition, hydration status, tissue properties, geometric changes between the abdominal muscles and the electrode, as well as external artifacts. The complex structure of the PFM further complicates isolated measurement of individual muscles. Another limitation of the present study could be the relatively short observation period of the training effects. Extending the training duration and, in future studies, incorporating a follow-up assessment after a certain time interval would allow for verification of whether the observed effects are maintained in the longer term. Future research should consider complementing sEMG with modern measurement tools such as ultrasonography or elastography, which may provide a more comprehensive assessment of PFM function.

## 5. Conclusions

The implementation of pelvic floor muscle (PFM) training was associated with improved neuromuscular activity of the PFMs, as demonstrated by sEMG assessment. Training according to the BeBo^®^ concept led to an improvement in resting PFM tone in women after cesarean delivery (CD) and in contraction speed in both study groups. In contrast, BeBo^®^ training supported by EMG-biofeedback proved more effective than BeBo^®^ training alone, as it improved the aforementioned variables as well as resting tone in women after vaginal delivery (VD), maximal contraction, and endurance during a 1 min sustained contraction in both training groups.

## 6. Practical Implications

1. The addition of EMG-biofeedback to BeBo^®^-based PFM training provides greater benefits across most analyzed variables compared with training without biofeedback. Therefore, the permanent inclusion of EMG-biofeedback in postpartum prophylaxis appears justified for both VD and CD women.
2. In physiotherapeutic management of women after CD, emphasis should be placed on relaxation exercises for the PFMs, whereas in women after VD a higher number of strengthening exercises is recommended.
3. This project demonstrated that even a single sEMG assessment procedure was associated with a reduction in PFM resting EMG amplitude in women after CD, suggesting the achievement of PFM relaxation.

## Figures and Tables

**Table 1:** Characteristics of the participants.

Group	Age [years]		Body Mass [kg]		Height [cm]		BMI [kg/m^2^]	
	$\overset{-}{x}$ ± SD	*p*-Value	$\overset{-}{x}$ ± SD	*p*-Value	$\overset{-}{x}$ ± SD	*p*-Value	$\overset{-}{x}$ ± SD	*p*-Value
I	28.01 ± 2.18	0.99	69.42 ± 8.18	0.99	167.35 ± 6.90	0.99	24.73 ± 1.94	0.99
II	29.01 ± 2.32		66.61 ± 8.09		164.35 ± 6.27		24.66 ± 2.57	
III	28.98 ± 2.16		66.30 ± 8.95		165.70 ± 6.01		24.07 ± 2.30	
IV	29.68 ± 1.57		71.38 ± 9.90		167.85 ± 4.59		25.32 ± 3.21	
V	29.40 ± 1.50		68.12 ± 8.83		166.85 ± 4.55		24.45 ± 2.88	
VI	29.01 ± 1.50		65.37 ± 8.22		162.80 ± 5.12		24.66 ± 2.88	

kg—kilogram, cm—centimeter, m^2^—square meter, BMI—Body Mass Index, Mean—mean value, SD—standard deviation, *p*—*p*-value for ANOVA test.

**Table 2:** Descriptive statistics and results of repeated measures ANOVA for mean minimum pelvic floor muscle tension (MinPFM) during 1 min rest.

Variable	Group	T1		T2		ES	Effect: D F *p* $\eta^{2}$	Effect: Tr F *p* $\eta^{2}$	D x Tr F *p* $\eta^{2}$	Effect: T F *p* $\eta^{2}$	T x D F *p* $\eta^{2}$	T x Tr F *p* $\eta^{2}$	T x D x Tr F *p* $\eta^{2}$	Post hoc Pre vs. Post *p*	T2 Group VD	T2 Group CD
		$\overset{-}{x}$ ± SD	%MVC	$\overset{-}{x}$ ± SD	%MVC											
MinPFM [µV]	I	5.18 ± 4.31	15%	3.43 ± 2.56	7%	−0.48	**24.533** **<0.001** **0.205**	1.195 0.307 0.025	0.306 0.737 0.006	**43.217** **<0.001** **0.313**	**6.134** **0.015** **0.061**	2.095 0.129 0.042	4.260 0.048 0.045	NS	I–III	IV–VI
	II	5.22 ± 3.13	16%	3.43 ± 2.13	8%	−0.65								NS	NS	0.041
	III	5.26 ± 3.26	15%	4.46 ± 3.01	12%	−0.25								NS	II–III	V–VI
	IV	11.51 ± 10.12	13%	6.56 ± 6.55	7%	−0.57								<0.001	NS	0.018
	V	10.82 ± 5.7	12%	6.01 ± 4.1	6%	−0.95								<0.001	I–II	IV–V
	VI	11.24 ± 6.4	14%	10.64 ± 6.84	12%	−0.09								NS	NS	0.049

T1, T2—time points: before therapy (1), after therapy (2), mean—average value, SD—standard deviation, %MVC—percentage of maximal voluntary contraction, MinPFM—mean minimum pelvic floor muscle tension, µV—microvolts, ES—effect size, SN—vaginal delivery, CC—cesarean section, NS—non-significant differences, D—delivery, T—time, Tr—training.

**Table 3:** Descriptive statistics and results of repeated measures ANOVA for parameters measured during 2 s phasic contractions.

Variable	Group	T1		T2		ES	Effect: D F *p* $\eta^{2}$	Effect: Tr F *p* $\eta^{2}$	D x Tr F *p* $\eta^{2}$	Effect: T F *p* $\eta^{2}$	T x D F *p* $\eta^{2}$	T x Tr F *p* $\eta^{2}$	T x D x Tr F *p* $\eta^{2}$	Post hoc Pre vs. Post *p*	T2 Group VD	T2 Group CD
		$\overset{-}{x}$ ± SD	%MVC	$\overset{-}{x}$ ± SD	%MVC											
MEAN MVC [µV]	I	28.57 ± 19.57	82%	35.11 ± 31.38	74%	0.25	**52.615** **<0.001** **0.359**	0.307 0.737 0.006	0.218 0.805 0.005	**3.159** **0.079** **0.033**	**0.107** **0.745** **0.001**	1.527 0.223 0.031	0.129 0.880 0.003	-		
	II	27.78 ± 14.56	83%	35.61 ± 19.06	81%	0.45								-		
	III	30.47 ± 20.57	86%	32.94 ± 18.87	87%	0.12								-		
	IV	66.76 ± 45.82	77%	73.54 ± 35.67	78%	0.16								-		
	V	73.59 ± 38.53	84%	86.01 ± 42.9	83%	0.30								-		
	VI	69.47 ± 42.07	84%	73.58 ± 36.18	93%	0.24								-		
MVC [µV]	I	34.64 ± 22.77	-	47.63 ± 64.93	-	0.26	**47.554** **<0.001** **0.336**	0.036 0.964 0.001	0.114 0.893 0.002	1.630 0.205 0.017	0.062 0.804 0.001	1.783 0.174 0.037	0.311 0.734 0.007	-		
	II	33.56 ± 17.63	-	43.75 ± 22.64	-	0.49								-		
	III	35.31 ± 22.85	-	37.88 ± 22.66	-	0.11								-		
	IV	86.36 ± 56.18	-	94.4 ± 52.99	-	0.14								-		
	V	87.91 ± 44.01	-	104.23 ± 51.6	-	0.33								-		
	VI	82.59 ± 46.42	-	85.15 ± 38.16	-	0.06								-		
MVC/MinPFM	I	958 ± 736	-	1588 ± 1584	-	0.50	1.900 0.171 0.020	0.717 0.491 0.015	0.181 0.835 0.004	**26.072** **<0.001** **0.217**	0.931 0.337 0.010	**4.101** **0.020** **0.080**	0.272 0.763 0.006	**0.045**	I–III	IV–VI
	II	851 ± 618	-	1901 ± 1900	-	0.73								**0.001**	NS	NS
	III	1070 ± 748	-	1199 ± 1030	-	0.14								NS	II–III	V–VI
	IV	1123 ± 823	-	2343 ± 2024	-	0.77								**0.001**	NS	**0.048**
	V	1200 ± 1256	-	2386 ± 1767	-	0.76								**<0.001**	I–II	IV–V
	VI	1050 ± 1027	-	1398 ± 1446	-	0.26								NS	NS	**0.049**
TBP [%]	I	45.50 ± 20.36	-	34.15 ± 12.71	-	−0.66	1.987 0.162 0.021	0.075 0.928 0.002	0.155 0.856 0.003	**17.258** **<0.001** **0.157**	0.162 0.689 0.002	**4.192** **0.042** **0.097**	0.026 0.974 0.001	**0.011**	I–III	IV–VI
	II	46.13 ± 15.32	-	32.97 ± 12.86	-	−0.91								**0.005**	**0.049**	NS
	III	43.03 ± 15.52	-	37.81 ± 13.59	-	−0.35								NS	II–III	V–VI
	IV	40.85 ± 11.77	-	32.46 ± 15.02	-	−0.61								**0.042**	**0.037**	**0.042**
	V	40.72 ± 16.09	-	30.58 ± 14.02	-	−0.66								**0.026**	I–II	IV–V
	VI	40.10 ± 13.95	-	36.84 ± 14.14	-	−0.23								NS	NS	**0.049**
MinPFM [µV]	I	5.43 ± 3.68	16%	4.54 ± 2.21	10%	−0.29	**17.551** **<0.001** **0.159**	1.000 0.372 0.021	0.100 0.905 0.002	**25.546** **<0.001** **0.215**	2.774 0.099 0.029	0.024 0.976 0.001	1.026 0.362 0.022	-		
	II	5.10 ± 2.80	15%	3.86 ± 1.95	9%	−0.51								-		
	III	5.89 ± 3.88	17%	4.61 ± 2.24	12%	−0.39								-		
	IV	10.15 ± 8.26	12%	6.55 ± 5.32	7%	−0.51								-		
	V	9.86 ± 6.17	11%	6.58 ± 4.6	6%	−0.59								-		
	VI	10.16 ± 6.3	12%	9.34 ± 7.21	11%	−0.12								-		

T1, T2—time points: before therapy (1), after therapy (2), mean—average value, SD—standard deviation, %MVC—percentage of maximal voluntary contraction, µV—microvolts, ES—effect size, MEAN MVC—mean peak amplitude during contraction phase, MVC—maximum amplitude value obtained during maximal contraction, MVC/MinPFM—ratio of maximum amplitude during maximal contraction to minimum value at rest, TBP—ratio of time from onset to peak (rise time) to total contraction duration, MinPFM—mean amplitude at rest between maximal contractions, SN—vaginal delivery, CC—cesarean section, NS—non-significant differences, D—delivery, T—time, Tr—training.

**Table 4:** Descriptive statistics and results of repeated measures ANOVA for parameters measured during 10 s tonic contractions.

Variable	Group	T1		T2		ES	Effect: D F *p* $\eta^{2}$	Effect: Tr F *p* $\eta^{2}$	D x Tr F *p* $\eta^{2}$	Effect: T F *p* $\eta^{2}$	T x D F *p* $\eta^{2}$	T x Tr F *p* $\eta^{2}$	T x D x Tr F *p* $\eta^{2}$	Post hoc Pre vs. Post *p*	T2 Group VD	T2 Group CD
		$\overset{-}{x}$ ± SD	%MVC	$\overset{-}{x}$ ± SD	%MVC											
Amplitude [µV]	I	15.20 ±10.09	44%	17.06 ±10.19	36%	0.18	**33.986** **<0.001** **0.263**	0.046 0.955 0.001	0.007 0.993 0.000	0.889 0.348 0.009	0.020 0.887 0.000	2.336 0.102 0.047	0.526 0.593 0.011	NS	I–III	IV–VI
	II	15.30 ± 8.35	46%	18.28 ±13.81	42%	0.26								NS	NS	NS
	III	15.07 ±11.53	43%	16.25 ±12.27	43%	0.10								NS	II–III	V–VI
	IV	34.54 ±27.43	40%	37.51 ±28.76	40%	0.10								NS	NS	NS
	V	34.42 ±19.25	39%	41.67 ±24.83	40%	0.32								**0.032**	I–II	IV–V
	VI	34.63 ± 24.3	42%	35.56 ±18.26	42%	0.04								NS	NS	NS
MinPFM [µV] [-]	I	5.77 ± 3.42	17%	5.2 ± 3.04	11%	−0.17	**23.524** **<0.001** **0.198**	0.748 0.476 0.016	0.053 0.948 0.001	**11.572** **0.001** **0.109**	1.627 0.205 0.017	0.476 0.623 0.010	0.051 0.950 0.001			
	II	6.01 ± 3.47	18%	4.69 ± 2.6	11%	−0.42										
	III	6.08 ± 3.73	17%	6.06 ± 4.07	16%	−0.01										
	IV	10.79 ± 8.78	12%	9.43 ± 6.53	10%	−0.24										
	V	10.88 ± 4.70	12%	7.97 ± 4.23	8%	−0.64										
	VI	11.51 ± 6.43	14%	9.95 ± 4.98	12%	−0.27										

T1, T2—time points: before therapy (1), after therapy (2), mean—average value, SD—standard deviation, %MVC—percentage of maximal voluntary contraction, µV—microvolts, ES—effect size; amplitude—mean amplitude during contractions, MinPFM—mean amplitude at rest between 10 s contractions, SN—vaginal delivery, CC—cesarean section, NS—non-significant differences, D—delivery, T—time, Tr—training.

**Table 5:** Descriptive statistics and results of repeated measures ANOVA for parameters measured during the 60 s endurance contraction.

Variable	Group	T1		T2		ES	Effect: D F *p* $\eta^{2}$	Effect: Tr F *p* $\eta^{2}$	D x Tr F *p* $\eta^{2}$	Effect: T F *p* $\eta^{2}$	T x D F *p* $\eta^{2}$	T x Tr F *p* $\eta^{2}$	T x D x Tr F *p* $\eta^{2}$	Post hoc Pre vs. Post *p*	T2 Group VD	T2 Group CD
		$\overset{-}{x}$ ± SD	%MVC	$\overset{-}{x}$ ± SD	%MVC											
MEDIAN [Hz]	I	66.78 ± 14.82	-	66.4 ± 12.85	-	−0.37	0.655 0.420 0.007	0.369 0.692 0.008	0.963 0.386 0.020	0.674 0.414 0.007	0.097 0.756 0.001	0.111 0.895 0.002	1.933 0.150 0.039	-	-	-
	II	66.28 ± 28.06	-	62.57 ± 20.86	-	−0.11								-	-	-
	III	63.64 ± 16.16	-	62.49 ± 15.26	-	0.06								-	-	-
	IV	65.91 ± 18.13	-	66.64 ± 13.77	-	−0.26								-	-	-
	V	62.43 ± 11.07	-	63.47 ± 11.96	-	−0.30								-	-	-
	VI	67.41 ± 16.14	-	67.78 ± 5.75	-	0.29								-	-	-
f III/II [-]	I	0.94 ± 0.05	-	1.00 ± 0.23	-	0.59	0.090 0.765 0.001	1.452 0.239 0.030	0.524 0.594 0.011	**13.826** **<0.001** **0.127**	0.028 0.868 0.000	**3.049** **0.045** **0.071**	0.021 0.979 0.000	NS	I–III	IV–VI
	II	0.91 ± 0.08	-	1.01 ± 0.08	-	1.59								**0.008**	NS	NS
	III	0.93 ± 0.08	-	0.94 ± 0.07	-	0.39								NS	II–III	V–VI
	IV	0.92 ± 0.07	-	0.99 ± 0.12	-	1.19								NS	**0.049**	**0.042**
	V	0.93 ± 0.10	-	1.03 ± 0.2	-	1.26								**0.006**	I–II	IV–V
	VI	0.94 ± 0.07	-	0.91 ± 0.06	-	0.44								NS	NS	**0.003**
Amplitude [µV]	I	13.10 ± 9.70	38%	14.85 ± 8.19	31%	0.19	**36.577** **<0.001** **0.280**	0.027 0.973 0.001	0.079 0.924 0.002	**5.156** **0.025** **0.052**	2.180 0.143 0.023	**3.680** **0.044** **0.064**	0.193 0.825 0.004	NS	I–III	IV–VI
	II	13.13 ± 7.59	39%	16.16 ± 8.78	37%	0.36								NS	NS	NS
	III	13.1 ± 10.61	37%	13.61 ± 11.76	36%	0.05								NS	II–III	V–VI
	IV	30.33 ± 22.37	35%	35.75 ± 30.38	38%	0.20								**0.024**	NS	NS
	V	29.57 ± 13.49	34%	36.84 ± 22.3	35%	0.39								**0.007**	I–II	IV–V
	VI	29.13 ± 15.20	35%	31.52 ± 17.3	37%	0.14								NS	NS	NS

T1, T2—time points: before therapy (1), after therapy (2), mean—average value, SD—standard deviation, %MVC—percentage of maximal voluntary contraction, µV—microvolts, ES—effect size, MEDIAN Hz—median frequency during contraction, f III/II—ratio of median frequency in interval III to interval II, amplitude—mean amplitude during contraction, SN—vaginal delivery, CC—cesarean section, NS—non-significant differences, D—delivery, T—time, Tr—training.

**Table 6:** Descriptive statistics and results of repeated measures ANOVA for mean minimum pelvic floor muscle tension (MinPFM [µV]) during 1 min rest.

**Variable**	**Group**	**T1**		**T2**		**ES**	Effect: D F *p* $\eta^{2}$	Effect: Tr F *p* $\eta^{2}$	D x Tr F *p* $\eta^{2}$	Effect: T F *p* $\eta^{2}$	T x D F *p* $\eta^{2}$	T x Tr F *p* $\eta^{2}$	T x D x Tr F *p* $\eta^{2}$	Post hoc Pre vs. Post *p*	T2 Group VD	T2 Group CD
		$\overset{-}{x}$ **± SD**	**%MVC**	**$\overset{-}{x}$ ± SD**	**%MVC**											
MinPFM[µV]	I	4.6 ± 18.32	13%	3.37 ± 18.92	8%	0.31	9.029 0.003 0.087	0.545 0.582 0.011	0.080 0.923 0.002	**26.170** **<0.001** **0.216**	0.953 0.331 0.010	0.171 0.843 0.004	0.108 0.898 0.002	-	-	-
	II	4.88 ± 14.57	15%	3.26 ± 8.43	7%	0.53								-	-	-
	III	4.62 ± 14.97	13%	4.16 ± 11.47	11%	0.14								-	-	-
	IV	7.82 ± 34.05	9%	5.84 ± 31.41	6%	0.26								-	-	-
	V	7.66 ± 18.06	9%	5.36 ± 17.77	5%	0.47								-	-	-
	VI	8.19 ± 22.87	10%	7.44 ± 6.96	9%	0.12								-	-	-

T1, T2—time points: before therapy (1), after therapy (2), mean—average value, SD—standard deviation; %MVC—percentage of maximal voluntary contraction, µV—microvolts, ES—effect size, SN—vaginal delivery, CC—cesarean section, D—delivery, T—time, Tr—training.

**Table 7:** Repeated measures analysis of variance comparing mean minimum pelvic floor muscle tension (MinPFM [µV]) during 1 min rest before and after the start of the sEMG procedure (MinPFM [µV]).

Variable	Group	Effect: D F *p* $\eta^{2}$	Effect: Tr F *p* $\eta^{2}$	D x Tr F *p* $\eta^{2}$	Effect: T F *p* $\eta^{2}$	T x D F *p* $\eta^{2}$	T x Tr F *p* $\eta^{2}$	T x D x Tr F *p* $\eta^{2}$	Post hoc Pre vs. Post *p*	T1 Group VD	T1 Group CD
MinPFM [µV]	I	**21.361** **<0.001** **0.164**	0.012 0.988 0.000	0.039 0.962 0.001	**42.491** **<0.001** **0.280**	**22.434** **<0.001** **0.171**	0.154 0.857 0.003	0.121 0.886 0.002	NS	I–III	IV–VI
	II								NS	NS	NS
	III								NS	II–III	V–VI
	IV								**<0.001**	NS	NS
	V								**<0.001**	I–II	IV–V
	VI								**<0.001**	NS	NS

MinPFM—mean minimum pelvic floor muscle tension, T1—time point: before therapy (1), µV—microvolts, SN—vaginal delivery, CC—cesarean section, NS—non-significant differences, D—delivery, T—time, Tr—training.

## Data Availability

All data generated or analyzed during this study are included in this published article. Further inquiries can be directed to the corresponding author(s).
